# Fatal thrombolysis-related intracerebral haemorrhage associated with amyloid-β-related angiitis in a middle-aged patient – case report and literature review

**DOI:** 10.1186/s12883-022-03029-x

**Published:** 2022-12-23

**Authors:** Zita Reisz, Claire Troakes, Laszlo K. Sztriha, Istvan Bodi

**Affiliations:** 1 Department of Clinical Neuropathology, King’s College Hospital NHS Foundation Trust, Denmark Hill, London, UK; 2 London Neurodegenerative Diseases Brain Bank, Department of Basic and Clinical Neuroscience, Institute of Psychiatry, Psychology and Neuroscience, King’s College London, London, UK; 3 Department of Neurology, King’s College Hospital NHS Foundation Trust, Denmark Hill, London, UK

**Keywords:** Case report, Amyloid-β-related angiitis, Amyloid-β, Cerebral amyloid angiopathy, Thrombolysis, Intracerebral haemorrhage

## Abstract

**Background:**

Amyloid-β-related angiitis (ABRA) is a rare complication of cerebral amyloid angiopathy, characterized by amyloid-β deposition in the leptomeningeal and cortical vessels with associated angiodestructive granulomatous inflammation. The clinical presentation is variable, including subacute cognitive decline, behavioural changes, headaches, seizures and focal neurological deficits, which may mimic other conditions. Here, we present a case with fatal thrombolysis-related haemorrhage associated with ABRA in a middle-aged patient.

**Case presentation:**

A 55-year-old man was admitted to hospital with sudden onset left-sided cheek, arm and hand sensory loss, blurred vision, and worsening headache, with a National Institutes of Health Stroke Scale (NIHSS) score of 3. An acute CT head scan showed no contraindications, and therefore the decision was made to give intravenous thrombolysis. Post-thrombolysis, he showed rapid deterioration with visual disturbances, headache and confusion, and a repeat CT head scan confirmed several areas of intracerebral haemorrhage. No benefit from surgical intervention was expected, and the patient died four days after the first presentation. Neuropathological examination found acute ischemic infarcts of three to five days duration in the basal ganglia, insular cortex and occipital lobe, correlating with the initial clinical symptoms. There were also extensive recent intracerebral haemorrhages most likely secondary to thrombolysis. Furthermore, the histological examination revealed severe cerebral amyloid angiopathy associated with granulomatous inflammatory reaction, consistent with ABRA.

**Conclusions:**

Presentation of ABRA in a middle-aged patient highlighted the difficulties in recognition and management of this rare condition. There is emerging evidence that patients with CAA may have increased risk of fatal intracerebral haemorrhages following thrombolysis. This may be further increased by a coexisting CAA-related inflammatory vasculopathy which is potentially treatable with steroid therapy if early diagnosis is made.

## Background

Cerebral amyloid angiopathy (CAA) is a frequent but often under-recognized disease in the elderly population, characterized by abnormal β-amyloid deposition mainly in the small and medium-sized vessels of the leptomeninges and of the cerebral cortex and the cerebellum. The prevalence of sporadic CAA increases with age, affecting approximately 30-50% of otherwise healthy older individuals, in which it might remain mild and often symptomless [1–4]. The strong association with dementia, particularly with Alzheimer’s disease (AD; ~ 80-90%), is well known and extensively investigated, and these cases often show more advanced pathology compared to patients without concomitant neurodegenerative disease [1, 4–6]. CAA may present with severe complications due to damage of the vessel wall (so-called CAA-related vasculopathy), including lobar macrohaemorrhages, cortical microbleeds and superficial cortical siderosis/focal convexity subarachnoid haemorrhages - sometimes without any previous clinical signs. Less frequently, the amyloid deposition induces obliterative vascular changes resulting in ischemic alterations in the form of cortical microinfarcts and white matter abnormalities [7–12].

Large cohort studies have found that CAA is the second most common cause of intracerebral haemorrhage following hypertensive disease, however, their clinical management is different, hence the importance of the accurate differential diagnosis before making any therapeutic decision [13, 14]. Despite many clinical trials, cerebral amyloid deposition, including CAA, is currently an unpreventable and untreatable disorder. Furthermore, CAA might even cause further challenge during treatment of other diseases due to the higher risk for haemorrhagic complications during thrombolysis, anticoagulation or anti-platelet therapies [4, 11, 15–17].

Recently, severe amyloid associated inflammatory complications were also described in the literature affecting only a subset of patients with a suspected autoimmune origin [18, 19]. Patients with so called cerebral amyloid angiopathy-related inflammation (CAA-ri) show a clinically distinct disease course usually presenting with subacute progressive behavioural symptoms, hallucinations, headaches, seizures and focal neurological deficits, which may mimic other disorders and cause a diagnostic challenge [19–21]. Asymmetrical, often mass-like white matter alterations, meningocortical macro-and microhaemorrhages and ischemic lesions on radiology, together with typical clinical presentation and presence of anti-amyloid antibody in cerebrospinal fluid further support the diagnosis [20, 22]. Brain biopsy demonstrating presence of CAA-related intramural lymphocytic inflammation is required for the diagnosis of CAA-ri [20, 21]. Based on distinctive morphology, amyloid β-related angiitis (ABRA) is characterized by an angiodestructive, often granulomatous inflammation showing some similarities with primary angiitis of the central nervous system (PACNS), however the latter typically occurs in younger individuals and is most likely idiopathic. Multinucleated giant cells with engulfed amyloid material within the cytoplasm are commonly seen and might help establish the morphological diagnosis of ABRA. Surgical biopsy specimens might be inconclusive due to segmental distribution of the inflammation making distinction between CAA-ri and ABRA difficult and in many cases only post-mortem examination could provide the definite diagnosis [23–25].

Here, we present the post-mortem findings of an ABRA case with unusual clinical presentation and further thrombolysis-related complications, highlighting the difficulties of recognition and management of this rare disease. This case has been presented as poster abstract at the 121st Meeting of the British Neuropathological Society [26].

## Case presentation

**Fig. 1:**
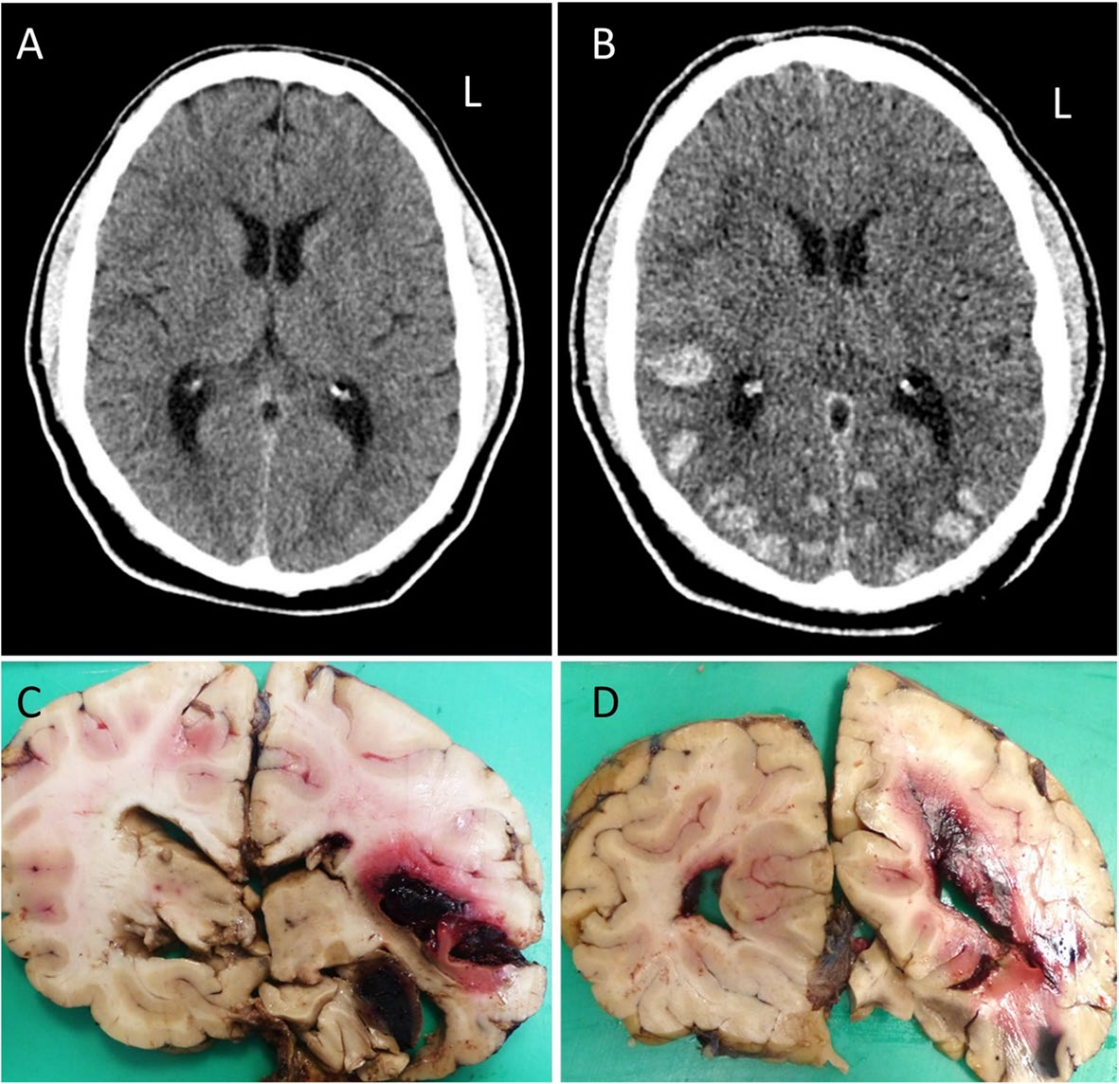
**a** Pre-thrombolysis non-contrast brain CT head scan demonstrating normal appearances, and in particular no evidence of haemorrhage. **b** After thrombolysis multiple foci of intracerebral haemorrhage with bilateral posterior hemispheric predominance are evident. **c**-**d** Macroscopic images of coronal brain sections showing multiple haemorrhages. There are recent intraparenchymal haemorrhages at the right insula and superior temporal gyrus (**c**) and the right occipital lobe (**d**). Extension to the subarachnoid space and the inferior (**c**) and posterior horns (**d**) is evident. Mild midline shift to the left is also present and the right uncus is partly necrotic (**c**)

A 55-year-old man was admitted to hospital as a stroke call, presenting with sudden-onset left sided cheek, arm and hand sensory loss, visual blurring, and worsening headache. The National Institutes of Health Stroke Scale (NIHSS) score was 3. His past medical history was significant for multiple co-morbidities including coronary artery bypass graft (CABG) surgery, hypertension, type 2 diabetes, and hypercholesterolemia. An urgent head CT excluded intracranial haemorrhage (Fig. 1A), and intravenous thrombolysis with alteplase was administered. Within 2 hours of thrombolysis, his features of visual disturbance and headache worsened, with increasing confusion. A repeat CT of the head showed multiple areas of intracerebral haemorrhage, mainly in the bilateral posterior regions (Fig. 1B). No benefit from neurosurgical intervention was expected. Despite intensive multiple organ support therapy, he deteriorated over his admission, developed chest infection, and died 4 days after the initial presentation. The case was referred to HM Coroner and detailed neuropathological examination was performed.

Informed consent was obtained from the relatives for research and medical education. Post-mortem examination confirmed marked cardiovascular disease, demonstrated by severe coronary artery sclerosis evident in the left main stem and right coronary arteries with associated critical stenosis (luminal diameter < 1 mm). The right coronary artery bypass graft was patent. There were no acute or healed myocardial infarcts. There was mild concentric left ventricular hypertrophy consistent with the history of hypertension. Examination of the lungs revealed an area of possible bronchopneumonia. Other internal organs showed no relevant alterations.

The brain was fixed in buffered formalin for 4 weeks and weighed 1466 g. On external examination, the brain was swollen and smooth with shallowed sulci and mild subarachnoid haemorrhages in the right anterior frontal pole, the right middle temporal and the right posterior parieto-occipital lobes. At the base of the brain both unci were bulged with prominent grooving, petechial haemorrhages on the right side and soft necrotic brain fragments on the left side. The cerebellar tonsils were also prominently grooved but without haemorrhages or softening. There was moderate calcifying atherosclerosis in the right vertebral artery without luminal narrowing. The rest of the basal arteries were normal with no evidence of thrombotic occlusion, vascular malformation or aneurysm.

Coronal sections of the cerebral hemispheres revealed mild midline shift to the left associated with compressed ventricles in the anterior and central parts (Fig. 1C). The right inferior horn and the posterior horns were filled with blood clots (Fig. 1C-D). There were multiple recent intracerebral parenchymal haemorrhages, various in size and widespread in distribution, affecting the right frontobasal area, the right temporo-occipital lobe, left superior parietal lobe, both posterior occipital lobe areas and the left cerebellar hemisphere. These haemorrhages probably occurred at the same time, and the atypical distribution suggested that they were secondary to the thrombolysis. Furthermore, a soft, cavitating area was identified in the subcortical white matter of the superior temporal gyrus and the insula slightly extending to the putamen, measuring 20x10x10mm. Timing was uncertain but appeared to be older than the haemorrhages, probably representing an ischaemic infarct. The hippocampi, the inferior part of the thalamus and the midbrain were haemorrhagic, soft and fragmented. Typical Duret’s haemorrhages were noted in the central part of the pons. The fourth ventricle contained a moderate amount of clotted blood. Summarizing the macroscopical findings, the direct cause of the death was given as brainstem herniation secondary to widespread recent intraparenchymal haemorrhages, probably related to thrombolysis. Alternative causes of the haemorrhages were also considered and further histological examination was performed.

**Fig. 2:**
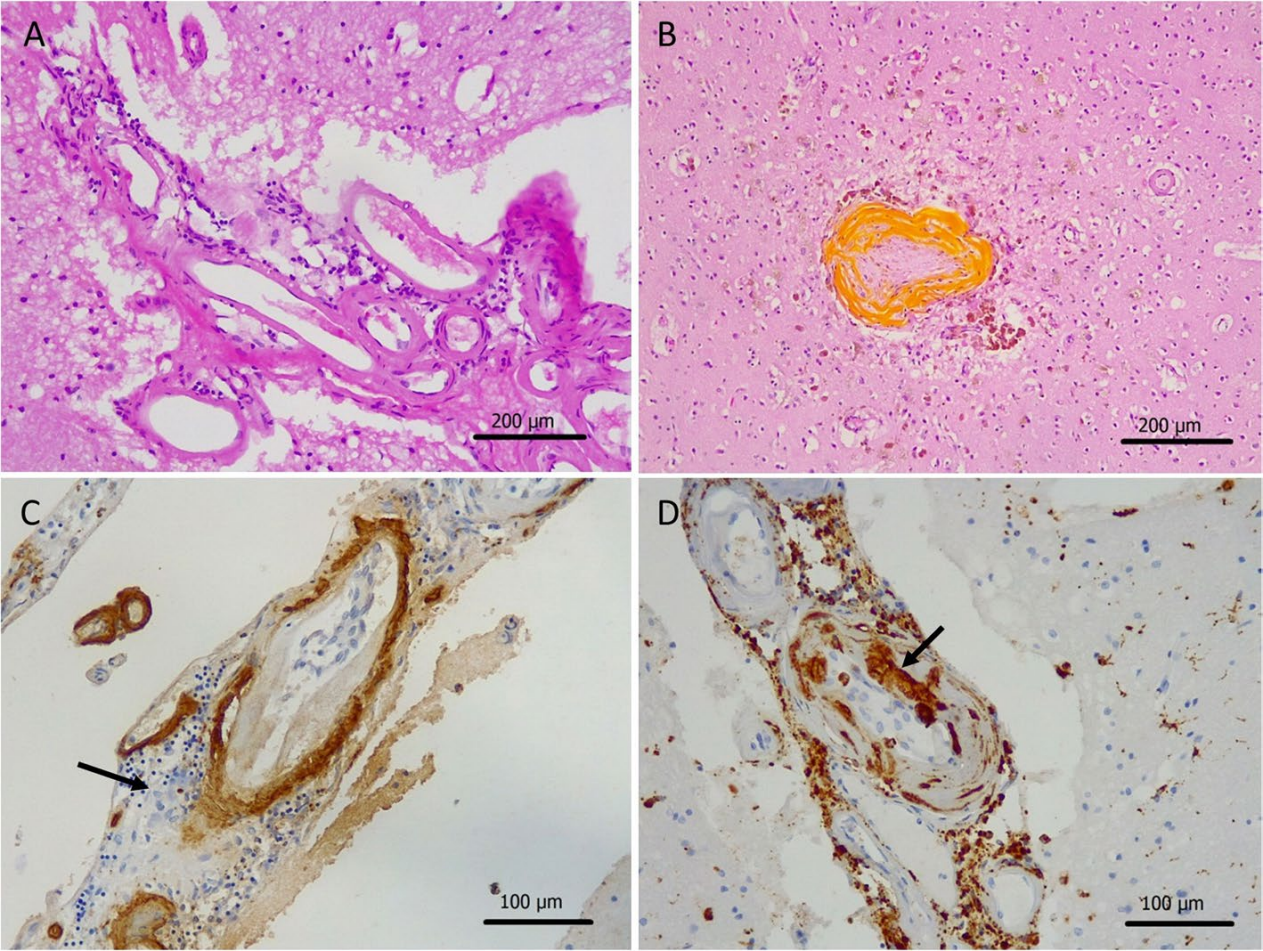
Histology of amyloid-β-related angiitis (ABRA). The leptomeningeal arteries show homogenous eosinophilic thickening, in keeping with marked cerebral amyloid angiopathy (CAA), surrounded by mild chronic inflammation by haematoxylin-eosin (HE) staining (**a**). Obliterated artery with haemosiderin deposition and old microinfarct in the left occipital lobe (**b**). Immunohistochemistry for amyloid-β confirms CAA and granulomatous inflammation with a multinucleated giant cell (arrow) is evident (**c**). Immunohistochemistry for CD68 demonstrates numerous perivascular macrophages and a giant cell invading the vessel wall (**d**)

Microscopic evaluation confirmed extensive recent intracerebral haemorrhages, most of them thought to be due to the thrombolysis therapy, surrounded by marked oedema and hypereosinophilic (red) neurons. Haemorrhages were also detected in the subarachnoid space and the ventricles, in keeping with secondary propagation of parenchymal haemorrhages. In addition, the histology examination also revealed severe CAA (Vonsattel grade 3), which was confirmed by Aβ immunostaining (Fig. 2). The CAA was particularly prominent in the occipital lobe and was associated with focal, perivascular and vaguely granulomatous inflammation, in keeping with ABRA (Fig. 2C-D). Further, probably subclinical, complications of CAA were noted in the left occipital lobe and in the white matter between the insular cortex and the putamen in form of old (at least 6 months) haemorrhagic microinfarcts (Fig. 2B). In addition, there were also two separate acute ischaemic lesions, estimated between 3 to 5 days old, in the right insular cortex and the basal ganglia and in the right occipital lobe, which might have caused the new onset neurological symptoms prior to hospitalisation. The parenchymal β-amyloid pathology was not significant, consisting of sparse mainly diffuse and fleece-like plaques throughout the neocortex. No characteristic cored plaques were identified and there were no neurofibrillary tangles by hyperphosphorylated tau, which excluded the possibility of underlying AD. Histological examination of the lung confirmed extensive bronchopneumonia which developed in the terminal stage and also contributed to death.

## Discussion and conclusions

ABRA is a rare life-threating CAA-related disorder typically affecting older individuals with a mean age at presentation of 67 years [3]. Risk factors specific for ABRA are not well-characterized, but the role of genetic factors, in particular certain allele variants of the ApoE gene, has been raised both in association with the disease itself and also with development of haemorrhagic complications. From the three most common isoforms, ApoE-ε4 and ApoE-ε2 were found to have an increased risk of CAA, while ApoE-ε2 was more frequently associated with development of fibrinoid necrosis and rupture of the affected blood vessels provoking intracerebral haemorrhages [27]. It appears that the dominating genotype among reported ABRA cases is the ApoE-ε4/ε4, while the ApoE-ε4/ε2 genotype shows less common occurrence [24, 27, 28]. Severe CAA affecting younger patients and associated with haemorrhagic complication should raise a concern of other rare hereditary factors, most frequently due to mutation of APP, PSEN1/2 or CYST C genes. Other familial amyloid angiopathies are typically not associated with intracerebral bleeds [4, 16].

The symptomatology of ABRA also significantly differs from sporadic non-inflammatory CAA, which usually presents with suddenly developing focal neurological symptoms due to lobar macrohaemorrhage, cortical microbleeding or ischaemic alterations. Patients with ABRA typically show subacute progressive symptoms including altered mental status, headaches, hallucination, seizures and only rarely focal neurological deficits. Stroke-like haemorrhagic complication is less common compared with CAA. These symptoms are not specific and can be observed in a wide range of other neurological disorders, such as infectious diseases (particularly progressive multifocal leukoencephalopathy), autoimmune encephalitis, neurosarcoidosis or other types of CNS vasculitides - including PACNS. Certain types of CNS malignancies should be also considered in the differential diagnosis, particularly CNS lymphomas, meningeal carcinomatosis and glial tumours with gliomatosis cerebri pattern [18].

The imaging and laboratory tests for CAA-ri and ABRA are not entirely specific, therefore establishing a definite diagnosis and making a therapeutic decision is often difficult. The neuroradiological appearances are highly variable and the findings required to be assessed in a complex clinical context. According to previous radiological studies on pathologically proven ABRA cases, T2-weighted and gradient recalled echo (GRE), or the more sensitive susceptibility-weighted imaging (SWI) appear to be the most useful MRI imaging to recognise the characteristic features, including the presence of lobar intraparenchymal haemorrhages, cortical microbleeds, microinfarcts and multifocal asymmetric patchy or confluent white matter hyperintensities [19, 25]. Amyloid deposition itself can be detected with Pittsburgh compound B using positron emission tomography, however this cannot further distinct between vascular or parenchymal localisation. Cerebrospinal fluid analysis is also a useful, although non-specific tool, usually demonstrating some degree of increased protein level associated with pleocytosis (usually lymphocytosis). Anti-amyloid-β autoantibody might be detected in the CSF from patients with CAA-ri, which further supports the autoimmune origin of this disease [29, 30]. The pathomechanism of these inflammatory reactions and the explanation of the rare occurrence among CAA cases is not well understood and requires further research.

CAA-ri and ABRA are theoretically treatable disorders often responding to steroids, therefore an early accurate diagnosis is crucial to prevent further complications. Unfortunately, in most cases the antemortem diagnosis is extremely challenging, particularly when the clinical course is not characteristic or the patient presents with rapidly progressive symptoms. The picture is further complicated by the fact that many of these patients suffer from multiple co-morbidities, which might be misleading and require careful risk assessment and complex therapeutic decisions. Currently, no widely accepted guidelines exist for the management of CAA and ABRA. Amyloid-related inflammation itself might show a good response to steroid therapy at least initially, but the primary vascular CAA pathology in the background will remain unchanged and carry a life-long high risk for further vascular events. Unfortunately, a small subset of CAA-ri cases will relapse despite adequate immunosuppression, potentially leading to a fatal outcome due to additional complications [21, 31]. The therapeutic strategy for CAA is mainly based on the management of comorbidities such as hypertension, cognitive follow-up and rehabilitation. Risk/benefit assessment of the anticoagulant therapy might be difficult in a complex case with several additional diseases, and there is emerging evidence that patients with CAA may have increased likelihood for intracerebral haemorrhage following thrombolysis [32–34]. Data are limited regarding the thrombolysis treatment in CAA-ri and ABRA, however it is expected that vascular inflammation might further increase the chance of therapy-related intracerebral bleeding.

In our case, many atypical factors presented together, leading to ABRA-related fatal outcome. The patient was quite young at presentation (55 years), which is not typical for ABRA or for CAA-related complications, and he had no clinical evidence of cognitive impairment or previous stroke which could have raised the possibility of an underlying sporadic CAA or AD. On the other hand, he had multiple significant comorbidities including severe coronary arteriosclerosis requiring previous CABG surgery, hypertension, type 2 diabetes and hypercholesterolemia, all of which are risk factors for ischaemic stroke, which might benefit from thrombolysis therapy. In the light of this, the chosen thrombolysis therapy seemed to be an appropriate decision, despite the patient’s low NIHSS score. The current guidelines do not contraindicate thrombolysis even in suspected CAA, and may underestimate the possibility of ABRA. The detailed post-mortem examination helped to explore the complex pathomechanism and connections behind the clinical course, the therapeutic complication and an unexpected rare underlying vascular disease (ABRA), raising many questions regarding the diagnostic approach for future cases of patients with suspected stroke.

## Data Availability

Access to the full data used in the study is available from the authors upon request.

## Abbreviations

AD: Alzheimer’s disease
ABRA: amyloid β-related angiitis
PACNS: primary angiitis of the central nervous system
CAA-ri: cerebral amyloid angiopathy-related inflammation
CABG: coronary artery bypass graft
Aβ: β-amyloid
CNS: central nervous system
GRE: gradient recalled echo
SWI: susceptibility-weighted imaging
MRI: Magnetic Resonance Imaging
NIHSS: National Institute of Health Stroke Scale
